# Exploring the Role of Cell-Free Nucleic Acids and Peritoneal Dialysis: A Narrative Review

**DOI:** 10.3390/genes15050553

**Published:** 2024-04-26

**Authors:** Niccolò Morisi, Grazia Maria Virzì, Marco Ferrarini, Gaetano Alfano, Monica Zanella, Claudio Ronco, Gabriele Donati

**Affiliations:** 1 Surgical, Medical, Dental and Morphological Sciences Department (CHIMOMO), University of Modena and Reggio Emilia, 41124 Modena, Italy; niccolo.morisi@unimore.it (N.M.);; 2 Department of Nephrology, Dialysis and Transplant, St. Bortolo Hospital, 36100 Vicenza, Italy; 3 IRRIV-International Renal Research Institute Vicenza-Foundation, 36100 Vicenza, Italy; 4 Nephrology Dialysis and Transplant Unit, University Hospital of Modena, 41124 Modena, Italy

**Keywords:** peritoneal dialysis, peritonitis, peritoneal aging, cell-free nucleic acids, cell-free DNA, cell-free RNA

## Abstract

Introduction: Cell-free nucleic acids (cf-NAs) represent a promising biomarker of various pathological and physiological conditions. Since its discovery in 1948, cf-NAs gained prognostic value in oncology, immunology, and other relevant fields. In peritoneal dialysis (PD), blood purification is performed by exposing the peritoneal membrane. Relevant sections: Complications of PD such as acute peritonitis and peritoneal membrane aging are often critical in PD patient management. In this review, we focused on bacterial DNA, cell-free DNA, mitochondrial DNA (mtDNA), microRNA (miRNA), and their potential uses as biomarkers for monitoring PD and its complications. For instance, the isolation of bacterial DNA in early acute peritonitis allows bacterial identification and subsequent therapy implementation. Cell-free DNA in peritoneal dialysis effluent (PDE) represents a marker of stress of the peritoneal membrane in both acute and chronic PD complications. Moreover, miRNA are promising hallmarks of peritoneal membrane remodeling and aging, even before its manifestation. In this scenario, with multiple cytokines involved, mtDNA could be considered equally meaningful to determine tissue inflammation. Conclusions: This review explores the relevance of cf-NAs in PD, demonstrating its promising role for both diagnosis and treatment. Further studies are necessary to implement the use of cf-NAs in PD clinical practice.

## 1. Introduction

Circulating cell-free DNA (cfDNA), the first of the cell-free nucleic acids (cfNAs) to be studied, was discovered by Mandel and Metais around 1948, but the scientific community failed to recognise the significance of this discovery for a long time [1]. In 1965, Bendich et al. proposed its role in cancer progression [2], followed by Tan et al. in 1966, linking elevated cfDNA levels to specific diseases like systemic lupus erythematosus [3]. In 1989, Stroun et al. demonstrated tumor-derived cfDNA [4]. At that time, the relationship between cfNAs and clinical status was clear. This was especially true for oncological diseases. However, the use of omics techniques remained outside clinical practice. Just in the last few decades, numerous omics techniques have been developed to gain a better understanding and elucidate the general pathways activated during various clinical conditions. The 1990s saw further advancements, with the discovery of tumor-specific mutations in cfDNA and identification of fetal cfDNA in maternal serum, leading to new diagnostic applications [5,6,7]. Further studies have highlighted the role of cfNAs in the diagnosis and monitoring of various pathological conditions, including the exploration of microRNA (miRNA) as a potential new target [8]. Particularly, the discovery of mitochondrial cfDNA expanded our understanding of its functions and clinical implications [9,10]. An integrated approach of multi-omic data could be helpful in discovering molecular dynamics and pathways implicated in the pathophysiology of human disease, thus leading to innovative strategies for their early detection, specific treatment, and effective prevention [11]. However, all of these technologies and approaches show some advantages and limitations [12]. In particular, omic technologies allow a comprehensive idea of the molecules, cells, and tissues. Numerous studies have shown that the integration of multi-omics data sets has been applied to a wide range of biological problems, helping to unravel the underlying mechanisms at the multi-omics level [13].

Peritoneal dialysis (PD) stands as a cornerstone in the management of end-stage renal disease (ESRD), offering a vital alternative to hemodialysis [14,15]. PD offers continuous clearance of toxins and excess fluids, potentially leading to better preservation of residual kidney function and improved patient outcomes [16]. However, PD presents challenges, including a significant risk of peritoneal infection, known as peritonitis, which leads to acute inflammation of the peritoneum [17]. Chronic inflammation, induced by continuous exposure of the peritoneum to high-concentration glucose dialysate fluids, contributes to the aging and remodeling of the peritoneal membrane [18]. This process results in a decline in peritoneal clearance function, ultimately leading to potential dropout from peritoneal dialysis.

This narrative review aims to describe the main features of cfNAs in peritoneal dialysis with a deeper focus on recent findings suggesting cfDNA as promising biomaker to evaluate peritonitis and peritoneal acute cell damage and to monitor the recovery process after it.

## 2. Literature Search Tools

Complete research in PubMed and Cochrane databases was carried out by these search strings: (“peritoneal membrane” OR “peritoneum”) AND (“omics” OR “systems biology” OR “multi-omics”) AND ((“microRNA” OR “miRNA”) OR (“cell-free DNA” OR “cfDNA”) OR (“mitochondrial DNA” OR “mtDNA”) OR (“cell-free nucleic acids” OR “cfNA”)) AND (“aging” OR “ageing”); (Peritonitis OR “Peritoneal Dialysis”) AND (“Cell-Free Nucleic Acids” OR “Cell-Free DNA”) NOT (Neoplasms OR “Kidney Transplantation” OR “Hemodialysis”). Furthermore, PubMed was used to identify narrative or systematic reviews and published using specific terms to elaborate and improve our results. The references of the retrieved papers were used to add more literature. We excluded case reports, case-series articles, and papers not in English. Furthermore, we did not include articles with only abstracts available. The first-choice criteria for article selection are: relevance of topic, evaluation of title and abstract, meta-analysis, clinical trial, original articles, guidelines repost, systematic review, and recent papers. Furthermore, we expanded bibliographic research using a narrative review of cell-free NAs in general.

## 3. Main Topics

### 3.1. Biological and Dynamics of Free Nucleic Acids

Various mechanisms permit the release of cfNAs in the extracellular compartment through necrosis, apoptosis, oncosis, NETosis suicide, and others [19,20]. Although it is unclear if these always retain biological function, cfNAs include several types of DNA (genomic DNA, mitochondrial DNA (mtDNA), microbial DNA) and RNA (miRNA, long non-coding RNA, circular RNA, YRNA and others) [21].

Various molecular biology techniques can be applied to analyze and quantify cfNAs in body fluids; we mentioned fluorescence, PCR (polymerase chain reaction), quantitative real-time PCR, droplet digital PCR, microarray, CGH array, sequencing, next-generation sequencing, and mass spectrometric-based analysis. Furthermore, for cfDNA characterization and for its origin determination, methylation analysis could be used (bisulfite or digested with methylation-sensitive restriction enzyme_MSRE) [22,23,24,25,26,27,28,29,30,31,32,33,34,35]. In particular, the process of extraction, purification, quantification, and origin determination is similar for serum, plasma, urine, and specific body fluids, such as peritoneal effluent.

In all processes, in vitro degradation of ctNAs and the additional release of contaminating genomic DNA must be carefully avoided. Unfortunately, the absence of harmonization of preanalytical protocol (such as different collection tubes, for example: specific tube for RNA, K_2_EDTA collection tube or specific Steck cell-free DNA blood collection tube for plasma cell-free DNA, specific different extraction and isolation protocols (automated extraction performed by different type of instruments with specific kits, two spin-column based, magnetic bead-based methods, and manual methods) and quantification methods, different centrifugation protocols, different storage and storage conditions) and molecular techniques used between different laboratories weakens the reproducibility of cfNAs results and hampers the cfNAs application [36]. For example, the appropriate extraction of cfNAs is critical to obtain optimum yields and purity. In addition to preanalytical variables, multiple biological factors such as insufficient reference materials and lack of quality controls confound the standardization of cfNAs analyses and limit validation of its clinical use [22,32,37].

#### 3.1.1. Cell-Free DNA

Human body fluids, such as plasma, serum urine, amniotic fluid, sputum, cerebrospinal fluid, peritoneal effluent etc., are deep sources of cell-free and eventually circulating nuclear acid material with peculiar properties. Cell-free DNA (cfDNA) refers to all non-encapsulated DNA fragments present in body fluid, both in physiological conditions as well as in pathological disorders, and they originate from the nucleus, mitochondria, diverse cell lines, tumors, all organs (including transplanted and damaged ones), fetuses, the environment, invading pathogens, and the microbiome [37].

cfDNA could have different sizes depending on the origin, the fragmentation process, and the release mechanism. In particular, cfDNA from the genome is characterized by 80–10,000 base-pair (bp), with normal peaks of 166 bp fragments, and mitochondrial cfDNA is defined by fragments of 20–100 bp [38].

In healthy and pathological conditions, the release processes from the intracellular section into the extracellular fluids can originate from the following situations: cellular death event, such as necrosis and apoptosis, active DNA release that occurs in viable cells (for example, exosome, microvesicles, or erythroblast enucleation), and foreign exogenous sources (bacterial and viral agents, microbiome) [20,39,40,41]. Furthermore, fetal cfDNA and cfDNA released from organ transplantations should be mentioned [42,43]. Despite its brief half-life, cfDNA is continuously recharged [37].

The notability of cfDNA is principally connected to its molecular features: cfDNA has multiple genetic characteristics and a specific epigenetic profile. Furthermore, the production of cfDNA is the demonstration of physiological and pathological biological activities and functions, such as immunomodulation, oncogenesis, and metastasis. The released mechanism, its origin, the involved process (physiological and/or pathological), and the presence of housekeeping genes or tissue-specific genes permit the correct identification and characterization of cfDNA [37].

In the last decade, the attention to cfDNA and the application of this tool has been expanded. The collection of cfDNA samples from human biological fluids is counted as relatively non-invasive and allows multiple and serial sample collections. It could be considered a sort of “liquid biopsy” because it provides diagnostic information without an invasive approach [44,45,46]. In addition, the cfDNA pool in body fluids offers real-time data on host and meta-genomic modifications that can be used for many different goals. Some examples of this fact are the use of cfDNA analysis in prenatal screening tests [47], in oncology for cancer diagnosis [48], and monitoring of organ transplant recipients [31,49].

Moreover, in addition to the information of genetic characteristics of cfDNA and its quantification, the peculiarity of methylation profiles, fragmentation patterns, and topological characteristics can also be applied for diagnostic advantage and utility [45]. In this context, cfDNA is rapidly transitioning from discovery research to an important tool in clinical decision-making in many fields with clinical applications in various diseases. In particular, cfDNA has an important role as a diagnostic or prognostic biomarker in many clinical fields such as oncology, cardiology, prenatal diagnosis, etc. Quantification and identification of cfDNA profiles are promising tools for monitoring different clinical conditions.

Amongst the various forms of cfDNA, recent attention has been drawn to cell-free mtDNA. Mitochondria, essential eukaryotic organelles with an α-proteobacterial origin, play crucial roles in energy production, biomolecule synthesis, cell proliferation, and apoptosis. Their circular, bacterial-like genome, approximately 16,569 bp long in human cells, exists in 100–10,000 copies [50]. Various forms of mtDNA are found in body fluids, including exposed fragments, vesicle-contained mtDNA, and extruded whole mitochondria [51]. Origins of mtDNA include autophagy, apoptosis, necrosis, active transport processes, and formation of NETs [9]. These mtDNA fragments, generally shorter than nuclear DNA fragments, range from 30–80 bp with peaks at 42–60 bp, though longer fragments up to 220 bp have been observed [52]. Microvesicles can transport both genomic and mitochondrial DNA, with potential implications for metabolic restoration or tumor cell activation and chemoresistance induction [53].

Changes in mtDNA copy numbers are associated with various human diseases, including cancer, neurodegenerative, and cardiovascular diseases [10,54]. The fragmentation pattern of mtDNA may serve as a potential biomarker, as shorter fragments are more likely released by tumor cells undergoing necrosis, compared to the apoptosis predominant in normal cells.

#### 3.1.2. Cell-Free RNA

The presence of RNA molecules in extracellular fluids signifies a significant aspect of intercellular communication. These RNAs might originate from either passive leakage due to cell death processes such as necrosis or apoptosis, or active secretion pathways involving membrane-bound vesicles or RNA–binding proteins [55,56]. The latter mechanisms are believed to be tightly regulated and energy-dependent processes.

While various types of cell-free RNAs (cfRNAs) have been identified, the focus primarily lies on non-coding cfRNAs, owing to their relative stability conferred by association with proteins or encapsulation within vesicles. The most studied cfRNA molecules are miRNA, which are small (18–23 nt) ssRNA molecules involved in posttranscriptional gene regulation. Studies have consistently shown differential expression patterns of miRNAs between individuals with diseases and healthy counterparts [57,58]. Alterations in miRNA expression are observed in various disorders including cancers, highlighting their diagnostic potential. Tumor-derived cf-miRNAs, packaged within vesicles, can modulate gene expression in recipient cells, influencing the tumor microenvironment and promoting tumorigenesis [59]. Moreover, miRNAs participate in diverse physiological processes beyond cancer, including maternal–fetal communication and host–parasite interactions [60]. Harnessing miRNAs as therapeutic targets holds promise for cancer therapy.

### 3.2. Cell-Free Nucleic Acids and Peritoneal Membrane (See Figure 1: The Role of Cell-Free Nucleic Acids in Peritoneal Dialysis)

The peritoneal membrane serves as the interface for peritoneal dialysis (PD), playing a crucial role in the exchange of solutes and fluids during the therapy. However, prolonged exposure to dialysis solutions can lead to structural and functional changes in the peritoneal membrane, potentially resulting in complications [61,62,63]. Early investigations were among the first to explore cfNAs as potential biomarkers for evaluating peritoneal membrane integrity and function in PD patients. These molecules offer valuable insights into cellular damage and turnover within the peritoneal cavity.

In the crossover study of Jernej P. et al. [64], the authors examine the presence of cfDNA in the peritoneal effluent of stable continuous ambulatory peritoneal dialysis (CAPD) patients for the first time. They observed a concentration range of 1.8–9.5 mg/L of cfDNA in overnight peritoneal effluent samples, indicating its detection within the peritoneal cavity. Furthermore, the study compared two different PD solutions—a conventional lactate-buffered acidic solution (solution D) and a novel bicarbonate/lactate-buffered neutral solution (solution P)—and identified a significant decrease in cfDNA appearance with the novel solution. These early findings suggest that cfNAs, particularly cfDNA, may serve as promising indicators of peritoneal membrane health and integrity in a small group of PD patients.

Recent research from Weifei W. [65] has focused on the intricate mechanisms underlying ultrafiltration failure, a critical complication of long-term PD with potentially fatal consequences. A notable study aimed to elucidate the role of circulating exosomal miRNAs in the context of ultrafiltration failure and its associated pathophysiology. Utilizing advanced techniques including transmission electron microscopy (TEM), nanoparticle tracking analysis (NTA), and small RNA sequencing, researchers isolated exosomes from peritoneal dialysis effluent (PDE) of patients experiencing ultrafiltration failure or success. This comprehensive analysis revealed differential expression of 70 miRNAs implicated in ultrafiltration, suggesting their potential role as biomarkers for this condition. The study underscores the significance of these circulating exosomal miRNAs as promising biomarkers, and opens avenues for exploring novel therapeutic targets to mitigate ultrafiltration failure in patients with end-stage renal disease. In light of these findings, different studies have been conducted, revealing that miRNAs seem to be linked to the pathogenesis of peritoneal fibrosis [66] until their maximum manifestation as encapsulated peritonitis [67].

Moreover, emerging research sheds light on the role of mitochondrial DNA (mtDNA) in perpetuating inflammation within the peritoneal cavity of PD patients. Released into the extracellular space following cell injury or death, mtDNA has been implicated in promoting inflammation in both clinical and experimental settings. Hence, sediment mtDNA was an independent predictor of technique survival [68]. However, the specific effects of peritoneal dialysate cell-free mtDNA on intraperitoneal inflammation and peritoneal solute transport rate (PSTR) in PD patients have remained elusive.

Apart from cf-mtDNA quantification, the degradation process from nuclease activity represents a potential biomarker. Since DNA inside mitochondria is protected from nuclease cleavage, degraded cf-mtDNA is a necrosis index in body fluids [21]. Compared to cfDNA specimens from serum and urine, with the first more protected by nucleosome binding and the latter more degraded, peritoneal cfDNA samples show a fragmentation pattern intermediate with approximately ¾ of fragments that exceed 100 bp [69]. Aside from that, no data are available on cf-mtDNA degradation, and clinical applications are yet to be explored.

A recent study by Xishao X. et al. [70] sought to address this knowledge gap by investigating the relationship between peritoneal effluent mtDNA levels and inflammatory markers, including IL-6, IL-17A, TNF-α, and IFN-γ, as well as PSTR in incident PD patients. The study included 189 PD healthy patients without peritonitis, with findings revealing a significant correlation between dialysate mtDNA levels and markers of intraperitoneal inflammation, including IL-6, TNF-α, and IFN-γ. Furthermore, the study identified a noteworthy association between dialysate mtDNA levels and PSTR, highlighting the potential impact of mtDNA on peritoneal membrane function. Importantly, after adjusting for multiple covariates, dialysate mtDNA levels remained independently correlated with IL-6 levels and PSTR. However, contrary to expectations, dialysate mtDNA levels were not found to be associated with patient survival.

In light of these recent studies investigating the role of cfNAs, it becomes increasingly evident that a comprehensive understanding of peritoneal membrane function and integrity is essential for optimizing patient outcomes. While traditional assessments of urea, creatinine, and glucose transport remain valuable, they may not capture subtle structural changes in the peritoneum that could impact long-term PD success. As such, modern PD practice should prioritize early detection of alterations in the peritoneal membrane, leveraging novel biomarkers and advanced diagnostic techniques. By doing so, nephrologists can better tailor treatment strategies and interventions to mitigate complications associated with peritoneal membrane dysfunction, ultimately enhancing the quality of care for PD patients.

### 3.3. Cell-Free Nucleids Acids and Peritonitis (See Figure 1: The Role of Cell-Free Nucleic Acids in Peritoneal Dialysis)

Peritonitis remains a significant complication in patients undergoing PD, leading to morbidity, mortality, and treatment failure [71]. The ability to rapidly diagnose peritonitis and monitor its course is paramount for timely intervention and improved patient outcomes. Cell-free nucleic acids have garnered attention as potential biomarkers for assessing peritonitis severity and monitoring treatment response in PD patients. Over the years, several studies have delved into the utility of cfDNA in evaluating peritonitis episodes in PD.

The first study that examined plasma cfDNA levels in PD patients experiencing peritonitis was in 2015 by Virzì G.M. et al. [72]. This investigation revealed a significant association between cfDNA plasma concentration and the time elapsed since the last peritonitis episode. The findings suggested that elevated cfDNA levels could serve as an indicator of acute peritoneal damage, shedding light on the repair process following peritonitis episodes. Building on this foundation, subsequent research investigated the variation of cfDNA levels in the peritoneal effluent of PD patients with and without peritonitis. This study demonstrated notably higher cfDNA levels in patients during peritonitis episodes compared to those without, suggesting that cfDNA could be a valuable marker for assessing the severity of peritoneal inflammation and damage during peritonitis episodes [73].

In 2016, another study [74] employed a murine model of septic peritonitis to explore the correlation between cfDNA levels that reflected the degree of tissue damage, and M1/M2 macrophage responses. The investigation revealed that cfDNA levels were closely associated with the balance between pro-inflammatory (M1) and anti-inflammatory (M2) macrophage responses, indicating cfDNA’s potential role as an indicator of the inflammatory status in peritonitis.

In a more recent study conducted in 2021, researchers investigated the utility of metagenomic sequencing of cfDNA extracted from peritoneal effluent in thirty-five PD patients with and without peritonitis. This cutting-edge approach enabled the detection and identification of various pathogens, even in cases where conventional cultures yielded negative results, and notably, even during ongoing antibiotic therapy. The study underscored the potential of metagenomic cfDNA sequencing as a sensitive and comprehensive method for diagnosing peritonitis in PD patients, demonstrating its efficacy in both culture-negative cases and those undergoing antibiotic treatment [69].

The current ISPD guidelines define that the average frequency of peritonitis per patient per year should not exceed the value of 0.4. This limit is often respected by centers, but this does not exclude the possibility of some patients exceeding it, and also experiencing recurrent episodes of peritonitis in a short time. Additionally, it is important to note that approximately 20% of peritonitis cases result in negative cultures, making it even more challenging to establish an effective treatment [71]. The innovative approach utilizing cfDNA appears promising for improving diagnostic efficacy in detecting peritonitis, particularly in cases where traditional cultures are negative or during antibiotic therapy, as well as for providing crucial prognostic insights into antibiotic therapy response. These findings suggest significant potential for clinical application of cfDNA sequencing in monitoring and managing peritonitis in peritoneal dialysis patients.

**Figure 1:**
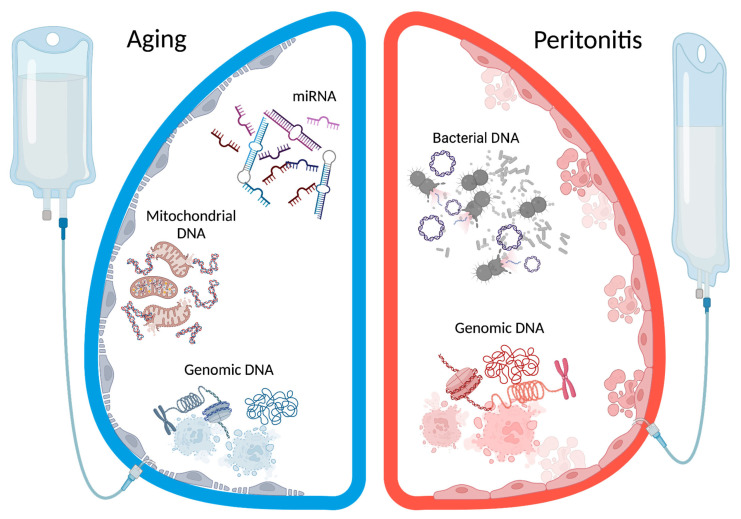
The role of cell-free nucleic acids in peritoneal dialysis (PD). (**left**), there is a graphical schematization of an ageing peritoneum. In that condition, there is a progressive loss of function of the membrane. In the peritoneal fluids, it is possible to detect cell-free DNA as a marker of PD solution toleration, and mitochondrial DNA as a marker of chronic inflammation. Moreover, it is possible to detect different miRNAs as biomarkers of predisposition and stage of fibrosis. (**right**), there is a graphical schematization of acute peritonitis. In this condition, peritoneal cells are damaged by inflammation and pathogens. This cell lysis causes a massive release of cell-free DNA that could be detected in peritoneal fluids, linked to damage status. Moreover, it is permitted to bacterial lysis of bacterial genomes as cell-free DNA both in culture-negative peritonitis and during antibiotic therapies.

## 4. Conclusions

In conclusion, the exploration of cell-free nucleic acids (cfNAs) in peritoneal dialysis (PD) offers promising insights into peritoneal membrane integrity, peritonitis severity, and treatment response (see Table 1). Recent studies have highlighted the diagnostic and prognostic potential of cfDNA, particularly in cases with negative culture results and ongoing antibiotic therapy. Additionally, investigations into microRNAs (miRNAs) and mitochondrial DNA (mtDNA) provide valuable insights into peritoneal aging and dysfunction. Integration of cfNA analysis into clinical practice holds the potential to optimize treatment strategies and improve patient outcomes in PD.

## 5. Future Direction

The field of cfNAs offers promising opportunities to improve clinical practice in peritoneal dialysis. A key perspective is the use of cfNAs as non-invasive biomarkers for early diagnosis and monitoring of patient conditions. With ongoing advances in sequencing technologies and bioinformatics, the identification of cfNA signatures specific to chronic kidney disease has the potential to improve diagnostic accuracy and patient prognosis. In addition, the integration of cfNAs into personalised medicine protocols could be a significant step forward. By using the unique molecular profiles reflected in cfNAs, healthcare providers could tailor therapies to the specific needs of each patient, optimising treatment efficacy and minimising adverse effects.

Apart from technical protocols, which cannot be standardised because they depend on different types of cfNAs, we could speculate on hypothetical clinical protocols. If the objective is the evaluation of the peritoneal membrane, we could propose to introduce the evaluation of significant cfNAs yearly and after each peritonitis, taking into account an appropriate time of negativisation to avoid acute phase contaminations. Instead, in the case of peritonitis, protocols are established to obtain the most rapid and specific information about it. In this context, cfNAs could play a primary role and be used as the first line in the diagnostic pathway. However, the real cost of these procedures and the need for more researchers lead us to propose these techniques as a second line, after a negative report of common cultural methods.

Beyond the diagnostic and therapeutic implications, cfNAs offer exciting opportunities to deepen our understanding of the underlying mechanisms of chronic kidney disease and to explore new therapeutic targets. By studying the interaction between cfNAs and cellular processes, researchers could identify new avenues for intervention and develop novel treatment modalities.

However, to fully realise the potential of cfNAs in peritoneal dialysis, several challenges need to be addressed, including standardising sample collection and analysis protocols, understanding biological variability, and validating findings in large, diverse patient cohorts. Collaborative efforts between multidisciplinary research teams, industry partners, and regulatory agencies will be essential to overcoming these obstacles and translating cfNA-based discoveries into tangible clinical benefits for patients undergoing peritoneal dialysis.

## Figures and Tables

**Table 1:** Studies characteristics and summary results. Legend: PD, peritoneal dialysis; Cf, cell-free; PDE, peritoneal dialysis effluent; #, data not found; PSDR, peritoneal solute transport rate; UF, ultrafiltration; DSR, dialytic sodium removal; α-ENaC, epithelial sodium channel α subunit; EPS, encapsulating peritoneal sclerosis; mtDNA, mitochondrial DNA.

	Cell-Free Nucleic Acid	Authors	Year	Population Under Study	Mean Cell-Free Nucleic Acid Amount	Results
Peritonitis	Cell-free DNA	Virzì G.M. et al [72].	2015	54 PD patients divided by acute, subacute and without peritonitis	16,025 GE/mL during peritonitis	Plasma Cf-DNA was correlated to other inflammation markers during peritonitis
		Virzì G.M. et al. [73]	2015	53 PD patients divided by peritonitis or not	25,523 GE/mL during peritonitis	PDE Cf-DNA increased during peritonitis and declined with restoration of the peritoneum
		Xin Y. et al. [74]	2016	20 mice with experimentally induced peritonitis divided by M1/M2 expression	Increase up to 1300 ng/mL during peritonitis in mice	Cf-DNA was a biomarker reflecting M1/M2 responses during inflammation
		Burnham P [69]	2021	33 PD patients divided by peritonitis or not	#	Cf-DNA was used to characterize both culture-positive and culture-negative peritonitis
Peritoneal membrane	Cell-free DNA	Jernej P. et al. [64]	2010	26 PD patients divided by PD solutions	5.4 microg/L in Dianeal solution	Cf-DNA may increase in a less biocompatible solution
	Cell-free RNA	Wu W. et al. [65]	2022	6 PD patients divided by UF failure	#	Identify 70 mi-RNAs involved in ultrafiltration failure.
		Tong Y. et al. [66]	2022	50 PD patients divided by PSDR, UF and DSR	#	MiR-432-5p has α-ENaC as a direct gene target and is correlated to PSDR, UF and DSR.
		Wu K.L. et al. [67]	2022	142 PD patients divided by EPS and no-EPS	#	Model-based miRNA PD effluents may discover the probability of EPS
	Mitochondrial DNA	Xie X. et al. [70]	2019	189 incident PD patients	4325 copies/μL	MtDNA was correlated to peritoneal inflammation but did not affect patient survival
		Than W.H. et al. [68]	2021	168 incident PD patients	#	Sediment mtDNA was correlated to peritoneal inflammation and technique survival

## Data Availability

No new data were created or analyzed in this study. Data sharing is not applicable to this article.
